# Low-cost stopped-flow and freeze-quench device for double mixing

**DOI:** 10.1016/j.ohx.2023.e00409

**Published:** 2023-03-01

**Authors:** Łukasz Bujnowicz, Rafał Pietras, Marcin Sarewicz, Artur Osyczka

**Affiliations:** Department of Molecular Biophysics, Faculty of Biochemistry, Biophysics and Biotechnology, Jagiellonian University, Kraków, Poland

**Keywords:** Stopped -flow, Freeze –quench, Kinetics of reactions, Sequential mixing, Enzymatic activity

## Abstract

Experiments based on fast reagent mixing and observation of reaction progress are considered a powerful tool for investigating the kinetics of chemical and enzymatic reactions. Various spectroscopic methods are used in monitoring the reaction progress, which require different sample preparation methods. Stopped-flow is the most widespread method, where the reaction in the liquid phase is observed by optical absorption spectroscopy. Albeit less popular, the freeze-quench method is also used, in which the reaction is rapidly stopped by freezing the sample at a given time point after the reaction onset. The frozen droplets of the sample are collected and measured at low temperatures in the solid state. Currently, many commercial solutions are available for freeze-quench or stopped-flow experiments, but despite the high price of the devices, most of these do not allow combining both these methods in a single experiment. This study presents a relatively simple solution that combines both these methods, thus making a complete study of chemical or enzymatic reactions possible. Besides, the presented solution enables sequential double mixing of reagents, which is generally problematic and cannot be done using commercial instruments.

## Graphical abstract

**Figure:**
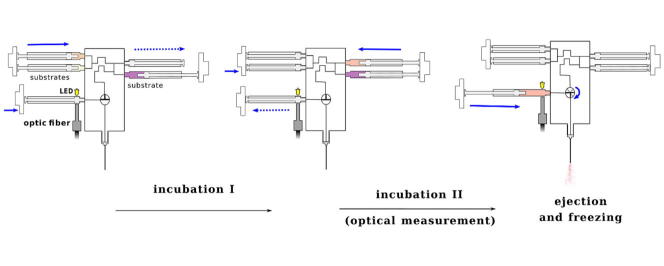

**Specifications table.**

**Table:** 

Hardware Name	Stopped-flow and freeze-quench device
Subject Area	• Engineering and Material Science • Chemistry and Biochemistry
Hardware Type	• Biological sample handling and preparation
Open Source License	**CERN Open Hardware License (OHL)**
Cost of Hardware	100 EUR
Source File Repository	https://doi.org/10.17632/kkf9w5p2fs.1

## Hardware in context

Studies of chemical and biochemical reactions require measuring transient nonequilibrium states in time that occur after the reagents are mixed until the steady state or equilibrium state is reached. In common laboratory techniques such as stopped-flow or freeze-quench, the reaction is usually initiated by ejecting reagents from syringes through a mixer into the reaction chamber. In the case of the stopped-flow technique, the reaction chamber also acts as an observation cell and the progress of the reaction can be continuously monitored by optical (absorption or fluorescence) spectroscopy[1]. In some cases, a detection of reaction intermediates requires cooling the sample to a low temperature.[2]. This is most common in studies of free radical enzymatic reactions, where radical detection is only carried out by low-temperature electron paramagnetic spectroscopy (EPR) since optical spectra are very weak or not detectable at all. In this case, the stopped-flow must be replaced by the freeze-quench method which allows trapping the intermediates of interest by freezing. However, this method is much more tedious as it requires measuring a series of samples frozen at particular time points that correspond to different times of the reaction. Combining the stopped-flow and freeze-quench methods would be of interest to obtain complementary information that can be traced simultaneously by absorption measurements and EPR (or any other low-temperature methods). Such an approach would be especially useful to study biological reactions catalyzed by enzymes.

The stopped-flow and freeze-quench methods can be used interchangeably with expensive commercially available devices, but it is usually difficult to find a device that would allow performing a unified experiment in which the reaction progress is monitored optically in the reaction chamber until its content is ejected into a cryogenic bath. Performing two independent experiments requires a higher amount of reagents, which may be a serious limitation—especially in the case of enzymatic studies—and may introduce additional uncertainty to the experiment. Because obtaining sufficient amounts of proteins from biological sources is often challenging and requires intensive and repetitive time- and cost-consuming purification steps, a reduction in sample consumption is considered a critical factor in the research. Furthermore, the results of two independent experiments do not always enable researchers to directly compare the results obtained using different spectroscopic methods and from two separate experiments. It is also worth noting that the use of both stopped-flow and freeze-quench techniques with commercially available devices involves high consumption of reagents. This is due to the need to wash all the pipelines and the reaction chambers to eliminate the remnants of the former reaction mixture, which should be carried out with a “new” reaction mixture that has to flow through the whole system, leading to a vast increase in sample loss. The sample loss is usually several times higher when the freeze-quench method is applied instead of stopped-flow because each time point of the reaction is registered as the beginning of a new reaction preceded by the washing of the system. Typically, stopped-flow and freeze-quench experiments involve simple mixing of only two reagents. However, some more complicated cases require sequential mixing of three solutions[3], for example, studies investigating chemical quenching of the reaction. Sequential mixing of reagents is generally difficult and requires sophisticated channel and chamber system architecture as well as careful programming of the mixing sequence[4]. Due to the much larger internal volume of such systems, reagent consumption is further increased in comparison with the case of simple mixing of two reagents.

The aim of the present study was to create a device combining freeze-quench and stopped-flow methods, so that they could be used simultaneously in an experiment. An additional objective of the study was to dramatically reduce the sample loss during sequential mixing of three solutions. The main purpose of this work is to develop an open-source and low-cost system capable of both modes, which is easy to create and modify.

## Hardware description

**Fig. 1:**
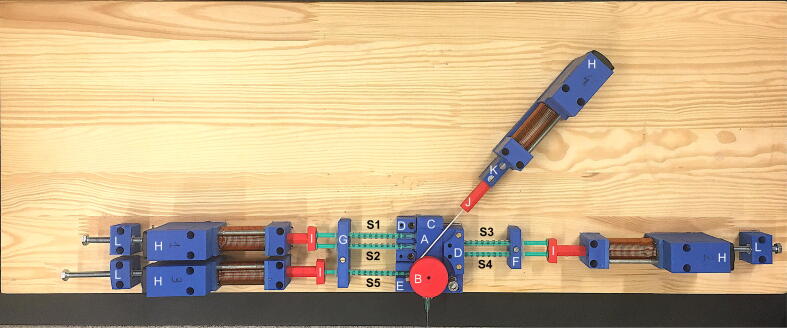
Picture of the device with depicted system elements: A—mixing block, B—valve wheel, C—mixer platform, D—two-syringe optic fiber holder, E—one-syringe optic fiber holder, F—two-syringe holder, G—three-syringe holder, H—coil of magnetic piston, I—piston of magnetic actuator, J—rod–string connector for magnetic actuator, K—rod holder, L—piston blockers. S1–S5 are the syringes numbered from 1 to 5.

The device described here consisted of a mixing block equipped with syringes, a support, and a system of magnetic actuators[5] that drive the pistons. A photograph of the entire assembly is presented in Fig. 1.

**Fig. 2:**
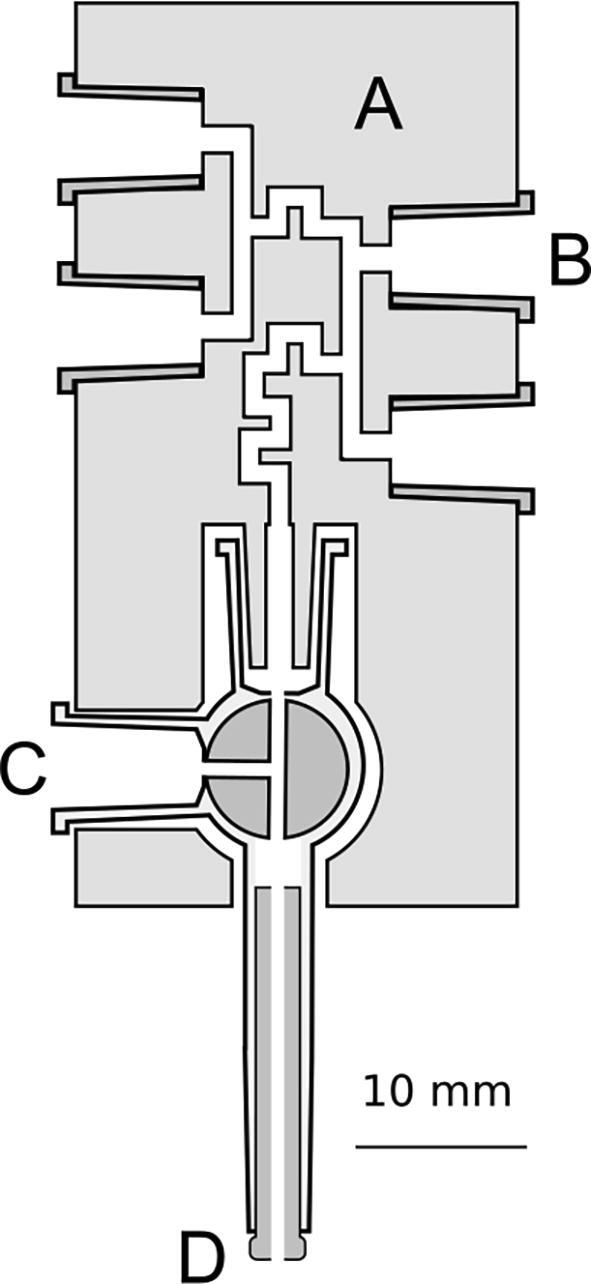
Cross-section of the mixing block: A—mixer, B—syringe sockets, C—three-way stopcock, D—outlet diameter reducer.

**Fig. 3:**
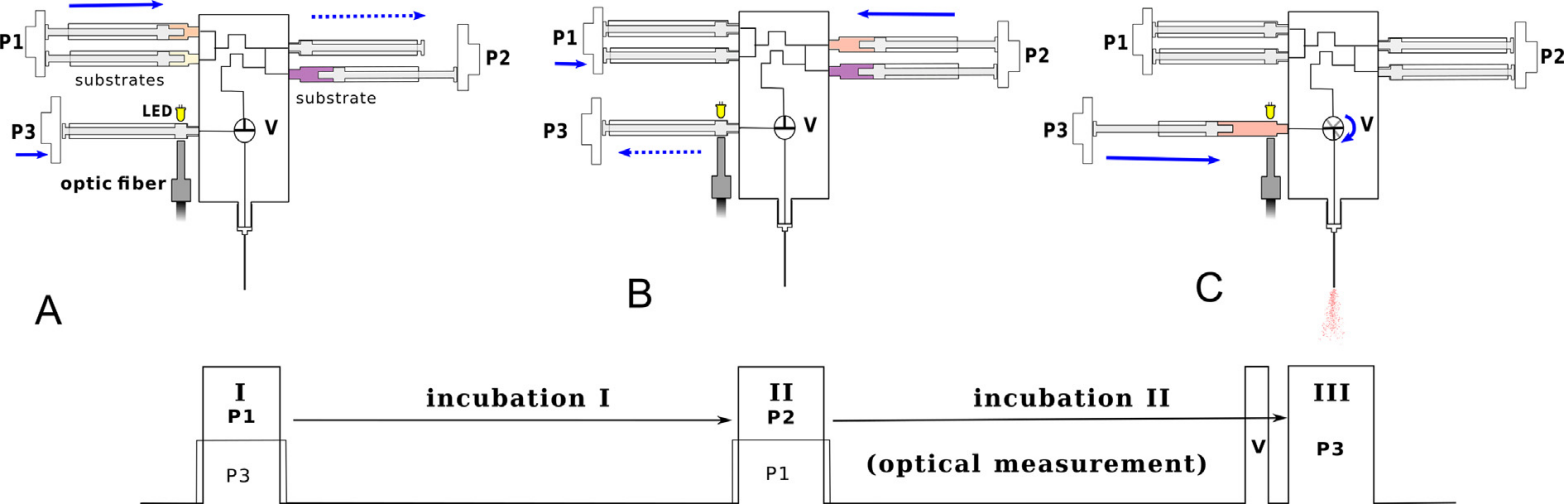
Scheme of a double-sequential-mixing experiment combining the stopped-flow and freeze-quench modes. The top panel shows the movement of the elements. The bottom panel shows the corresponding pulse sequence. A—the first mixing: the mixture is pushed into syringe 3 and the first incubation is started. B—the second mixing: the mixture incubated in syringe 3 is mixed with the third reagent. The second incubation and the optical measurement of the final mixture are started in syringe 5. C—the ejection of the final mixture after the second incubation to the cryogenic bath. The end of the optical measurement. P1–P2 are the pistons of the corresponding magnetic actuators, and V is the valve. Blue arrows indicate the movement of the piston/valve or the applied force.

The mixing block is the most important part of the device, which is based on a proprietary solution. It comprises a syringe, which serves as a reaction chambers of variable volume, and a valve switching the system between the stopped-flow and the freeze-quench mode. Due to the variable volume of the reaction chamber, the washing phase of the mixing sequence is eliminated, thereby reducing the amount of reagents needed for the experiment to a great extent. Besides, easy expansion of the system can be achieved using the solution based on the variable volume of the reaction chamber, which enables sequential mixing of the reactants. In a stopped-flow experiment, the reaction chamber is closed when mixing is complete and its volume is kept constant to avoid fluid fluctuations that may disturb reaction monitoring. In the freeze-quench mode, the system needs to be opened to eject the reaction mixture into the cryogenic bath. The connection between the reaction chamber and the valve that opens the system enables the ejection of the mixture. This way, the stopped-flow and freeze-quench techniques can be combined for a single experiment. The design uses the T-port rotary valve to allow easy and fast switching between three modes of operation, which can be useful for different types of experiments. The mixing block is a rectangular cube, within which there are connected channels for syringes inserted into the block and a rotary valve. The meandering parts of the channels act as mixers, and the turbulent flow inside these mixers ensures homogenization of the solutions. A cross-section through a mixing block is shown in Fig. 2. An exemplary experiment with stopped-flow and freeze-quench methods combined, where three reagents are mixed sequentially, is shown in Fig. 3.

The open-source multichannel pulse driver of magnetic actuator used to drive the syringes, pistons, and the valve used in this study has been previously described in Ref.[5]). The cited paper describes in detail the construction of the whole device necessary to drive syringes, including electronics, magnetic actuators, and software for programming the sequences with the user manual. While building the system described in Ref.[5], the pulse driver need not be modified; thus, it is not described in the present paper.

The developed system also includes a mixing block holder, adjustable plugs to prevent excessive syringe retraction, a syringe, an optic fiber, and actuator stabilizers. There is also a wheel to change the position of the valve through a string connected to one of the magnetic actuators.

Most of the elements of the device, apart from the magnetic actuator system, are designed in such a way that they can be manufactured on a standard 3D printer. The rest of the necessary items, such as a valve, syringes, and screws, can be purchased from local suppliers.

### Application of the device

The presented solution is dedicated to the investigation of chemical and biochemical reactions on the timescales of subseconds and seconds. It is worth mentioning that varying the volume of the reaction chamber, as shown here, solves some of the previously described problems; however, it is associated with some uncertainty about the incubation time therein. The range of this uncertainty is equivalent to the chamber filling time, which is in the order of several tens of milliseconds. Therefore, to investigate reactions with a time constant below 100 ms, this solution is not optimal. The device described in the present paper is not equally easy in handling as commercial devices but is useful for enzymatic investigations as it allows performing a multispectroscopic approach and saving reactants which are great advantages. Moreover, the low construction cost of the device and the possibility of customizing the mixing block make this device highly useful for laboratories dealing with the kinetics of enzymatic reactions.

The described device can be used in the following applications:

• stopped-flow
• freeze-quench
• quench-flow
• single and sequential mixing of reagents.
• a combination of all of the above

## Design files summary

Three download packages have been prepared:

The source code of the designed 3D printing is available as OpenSCAD files in the EJKL_mixer_openscad.zip package. The files provide information on mixer block, syringe and optic fiber holders, mixer platform, valve wheel, pistons, piston blockers, and outlet diameter reducer.

EJKL_mixer_stl.zip package contains the stl files of the described elements ready for 3D printing.

Pictures_movie.zip package contains all figures of the manuscript. It also has a record of tests of mixing described below.

All packages and files can be downloaded from the Mendeley repository, the links to which are given in Table 1. All files, except for the pictures_movie package, are also available at https://github.com/LukaszBujnowiczLab/EJKL_mixer.

**Table 1:** List of packages for download.

Design file name	File type	Open source license	Location of the file
EJKL_mixer_openscad.zip	.scad projects (3D printing or CNC milling)	**CERN OHL**	https://doi.org/10.17632/kkf9w5p2fs.1
EJKL_mixer_stl.zip	.stl files (3D printing or CNC milling)	**CERN OHL**	https://doi.org/10.17632/kkf9w5p2fs.1
Pictures_movie.zip	PNG, SVG, MP4	**CERN OHL**	https://doi.org/10.17632/kkf9w5p2fs.1

## Bill of materials summary

All the necessary names of the components and materials used to build the device, along with their prices, are given in Table 2. Links to online shops where the elements can be purchased are also presented in the table, but most of them are usually available at local markets. The elements required to build the magnetic actuators were omitted as they were already described in Ref.[5].

**Table 2:** Bills of materials.

Component	Number	Cost per unit, €	Total cost, €	Source of materials	Material type
Filament Noctuo ABS 1.75 mm, 0.75 kg	1	18.23	18.23	https://botland.com.pl	Plastic
Poxipol glue	1	3.38	3.38	https://botland.com.pl	Glue
Three-way stopcock KD-FLEX Standard Blue	1	0.15	0.15	https://medimall.pl	Plastic
Disposable needles length 120 mm	1	33.30	33.30	https://www.carlroth.com	Steel/plastic
Disposable syringe BBraun Injekt® F 1 mL	1	22.20	22.20	https://www.carlroth.com	Plastic
Wooden board 1.8 × 30 × 100 cm	1	12.92	12.92	https://www.obi.pl	Wood
Socket cap screw DIN 912 A2 M6 × 40	1	0.13	0.13	https://bejmet-nierdzewne.pl	Steel
Socket cap screw DIN 912 A2 M4 × 8	1	0.05	0.05	https://bejmet-nierdzewne.pl	Steel
Chipboard cuntersunk full thread DIN 7997 A2 Ø 3.5 × 30	4	0.03	0.12	https://bejmet-nierdzewne.pl	Steel
Chipboard cuntersunk full thread DIN 7997 A2 Ø 3.5 × 25	34	0.02	0.68	https://bejmet-nierdzewne.pl	Steel
Hexagon nut 3 DIN 6334 A2 M6	4	0.24	0.96	https://bejmet-nierdzewne.pl	Steel
Hexagon head screw DIN 933 A2 M6 × 80	3	0.16	0.48	https://bejmet-nierdzewne.pl	Steel
Phillips pan head screw DIN 7985 A2 M3 × 20	6	0.02	0.12	https://bejmet-nierdzewne.pl	Steel
Hexagon full nut DIN 934 A2 M 3	6	0.01	0.06	https://bejmet-nierdzewne.pl	Steel
LED 3 mm	5	0.13	0.65	www.tme.eu	Electronics
Female socket pin strips; DS1023-1*2S21	5	0.07	0.35	www.tme.eu	Electronics
		Total:	93.78		

## Build instructions

### 3D printing

Most of the elements were designed by 3D printing (Creality Ender-5 3D printer was used for this study) by applying the fused filament fabrication technology. ABS, which is a popular and durable material, is recommended as it allows slight corrections of a printed element to be made by means of acetone wash. However, materials other than ABS can also be used to build the device. Since printing with ABS requires a relatively high ambient temperature, the printer was shielded during the operation in order to increase the surrounding air temperature in the working volume with the bed temperature set to 100 °C. The temperature of the nozzle was set to 240 °C. The mixer, the valve wheel, the piston, the rod–string connector, the piston blocker rod, and the outlet diameter reducer should be printed with full filling. For preparing the rest of the elements, 50% of filling should be enough, provided that the wall thickness is at least 2 mm. The layer height was 0.06 mm for the outlet diameter reducer and 0.2 mm for other elements. The speed of the 3D printer head should be approximately 80 mm/s.

### Assembly of elements

#### Mixing block

**Fig. 4:**
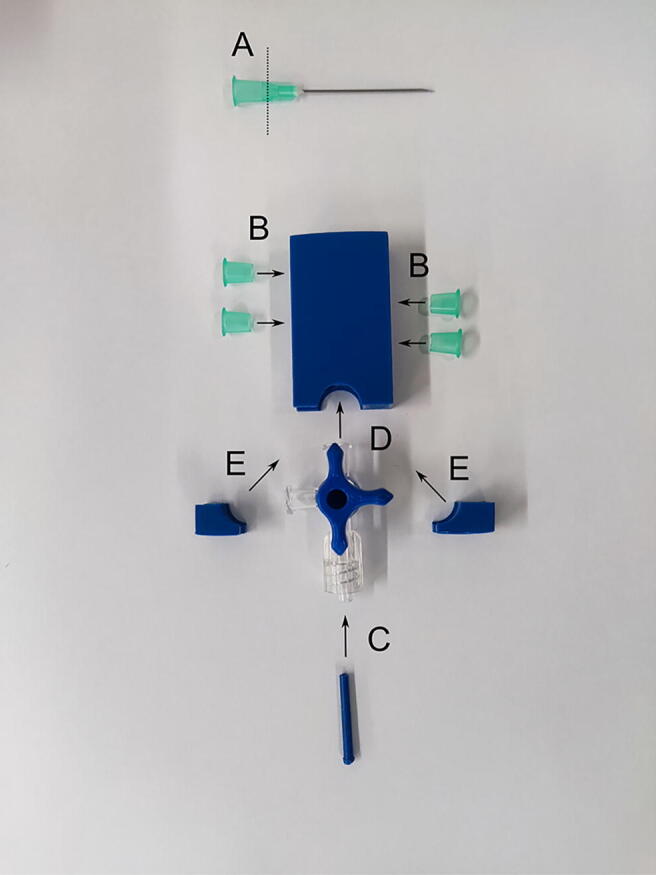
Assembly of the mixing block presented in alphabetical order: A—cut off the syringe socket from the disposable medical needle, B—insert the syringe sockets into the mixer, C—insert the reducer into the three-way stopcock outlet, C—paste the three-way stopcock into the mixer, D—paste the remaining fragments of the mixing block.

To assemble the mixing block, the mixer_body, the mixer_left_corner, the mixer_right_corner, and the outlet_diameter_reducer need to be printed in advance. Other components to be prepared are the three-way stopcock and sockets for the syringes that can be cut off with a knife from the disposable needle in the place indicated in Fig. 4. First, the sockets for the syringes have to be glued into the mixer.

A thick, sticky, and fast-setting epoxy resin is recommended for this purpose (POXIPOL® glue was used in this present study). Gluing should be carried out in accordance with the instructions provided by the glue vendor, ensuring that the channels in the mixer are not blocked. After the resin is hardened, the channels need to be checked for patency and the mixer tightness by pushing water through the mixer using syringes. If there are small blockages inside the mixer, obstructing the flow, or if there are visible microleaks between the print layers, acetone has to be pushed through the channels followed by ethanol (if the print is ABS). However, care should be taken not to dissolve the components of the mixer excessively. The diameter reducer needs to be checked to verify whether it fits in the outflow of the three-way stopcock. If there is difficulty with inserting it, the reducer should be rubbed with acetone-soaked paper to make its surface even. Then, the epoxy resin has to be applied to glue the mixer with the three-way stopcock and the corners of the mixing block. It is necessary to make sure that the gluing of the three-way stopcock to the outflow of the mixer is tight. After joining the pieces together, excess resin that leaked out has to be removed before it becomes hard.

### Mixer support, piston blocker, and syringe holders

**Fig. 5:**
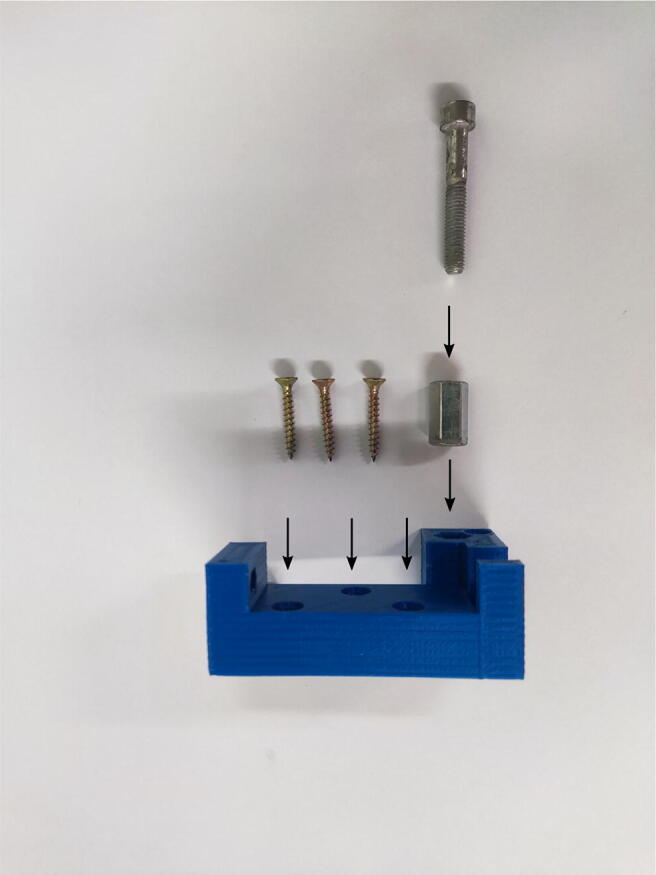
Assembly of the mixer platform.

**Fig. 6:**
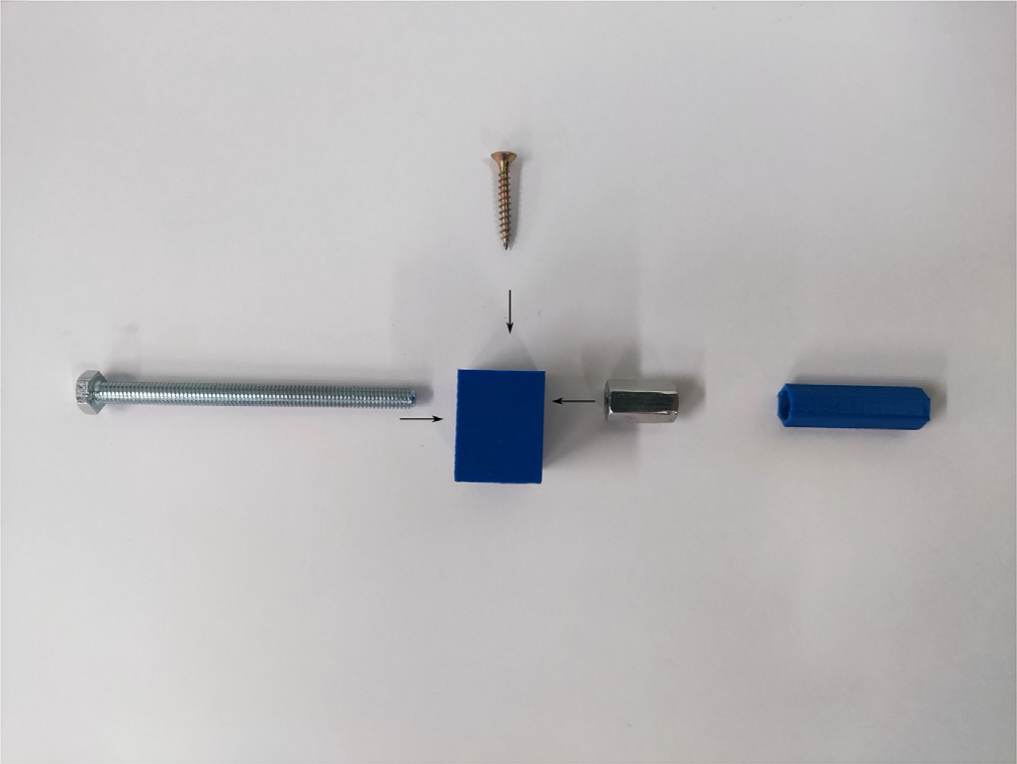
Assembly of the piston blockers.

The respective elements have to be printed and then the long M6 nut has to be placed in the dedicated space. If the dimensions of the hole are too small, the nut can be pressed with a vice. On the other hand, if nut is too loose, it can be glued with the resin. Then the M6 bolts need to be tightened into the nuts as shown in Fig. 5, Fig. 6. In the case of a piston blocker, a dedicated printed rod should be attached to the thread. Syringe holders consist of a lower part screwed to the board and an upper pressing syringe screwed with M3 screws to the nut placed on the bottom of the lower part. Wood screws should be placed into the appropriate holes in order to attach the elements to the board.

### Valve wheel

**Fig. 7:**
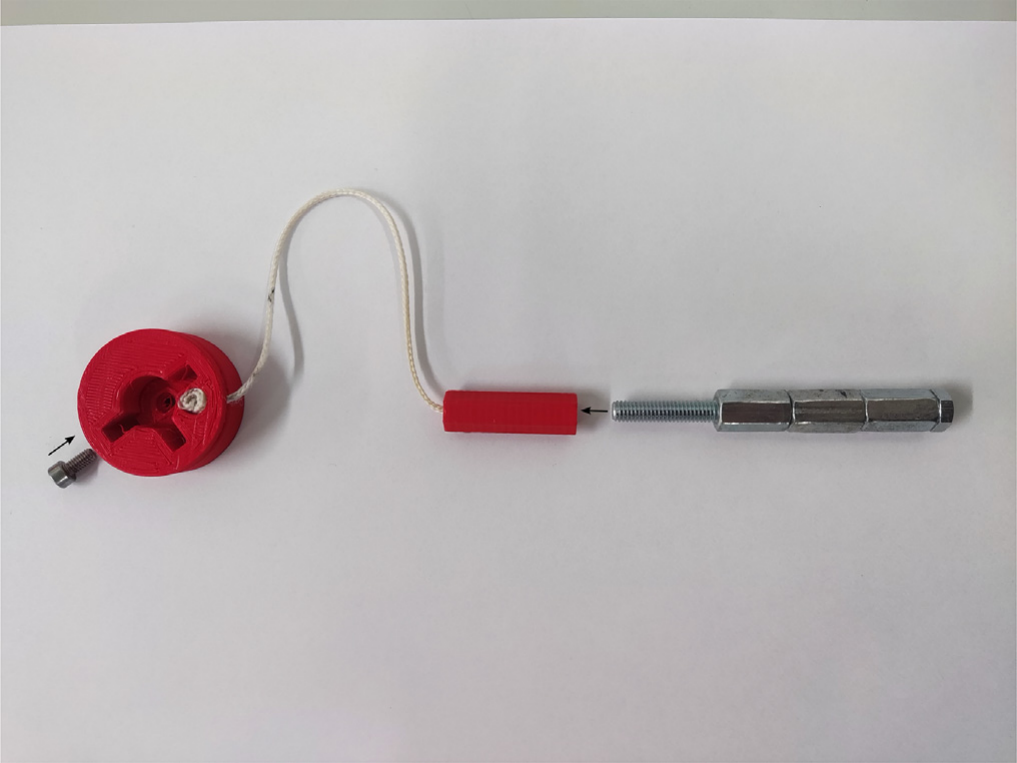
Assembly of the valve wheel system.

The printed wheel consists of a hole for a valve, two holes for a screw blocking the rotation, and a hole for hooking a string connected to the rod. In most of the experiments, the screw blocking the rotation (DIN 912 A2 M4 × 8) should be screwed in parallel to the long arm of the valve. A 15-cm-long nonstretchable string is attached to the wheel and rod using a simple knot. The thread of the metal core of the actuator should be screwed with the rod with the string instead of the piston. The assembling is shown in Fig. 7.

### Optic fiber holders

**Fig. 8:**
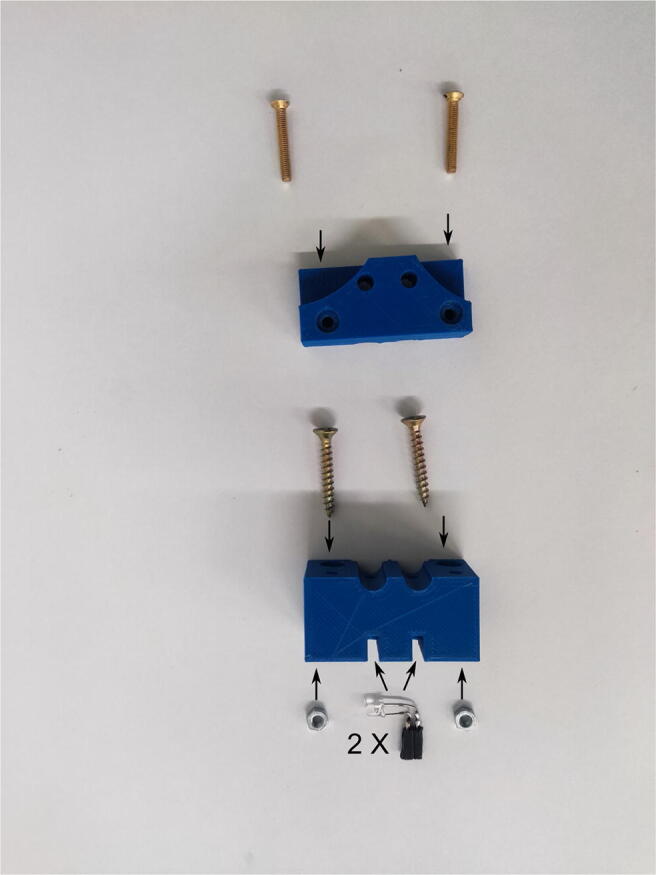
Assembly of the two-syringes optic fiber holder.

**Fig. 9:**
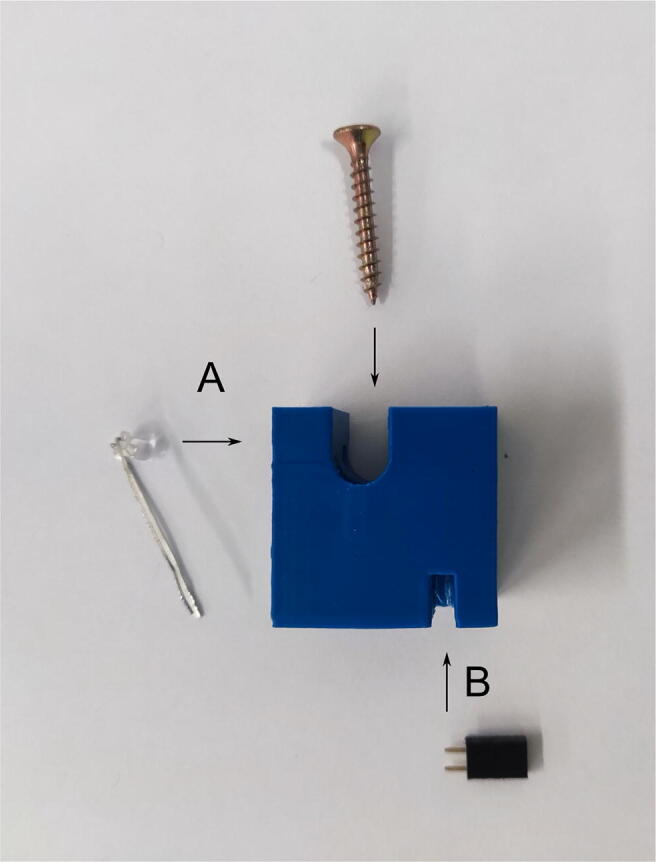
Assembly of the one-syringe optic fiber holder presented in alphabetical order: A—insert LED in the prepared hole, B—solder the pin socket to the diode.

The optic fiber holder for two syringes consists of top and bottom parts. In the bottom part, 3-mm LEDs are placed, which act as the light source (one diode per syringe). The spectrum of the diodes should be fit to the absorbance spectrum of the monitored compound. A white LED was used in this study. For two-syringe holders, the diodes should be soldered to the pin socket before they are inserted into the dedicated hole. The M3 nuts should be placed and fixed with a drop of glue in the dedicated hexagonal holes. The top part of the holder is fixed to the bottom part by two M3 screws. The assembling of the element is presented in Fig. 8. The holders are adapted to optic fibers of 4-mm diameter. If optic fibers of a different diameter are used, a correction in the respective openscad files has to be made. For one-syringe holders, the LED is on the side and the pin socket is on the bottom. The diode pins need to be bent as shown in Fig. 9. The pin socket should be soldered to the diode already placed in the holder.

### Magnetic actuator assembling

A detailed description of the winding of the coils, the assembling of the core, pistons, and holders, as well as the mounting of sockets is presented in Ref.[5]. The fastening screws should be inserted into the coil holder before the sockets are soldered to the coil wires. For this purpose, wood screws were used in this study. In the case of holders for the coil used to rotate the valve, 30-mm-long screws should be used, while for other coils 25-mm screws are used.

### Mounting on a board

**Fig. 10:**
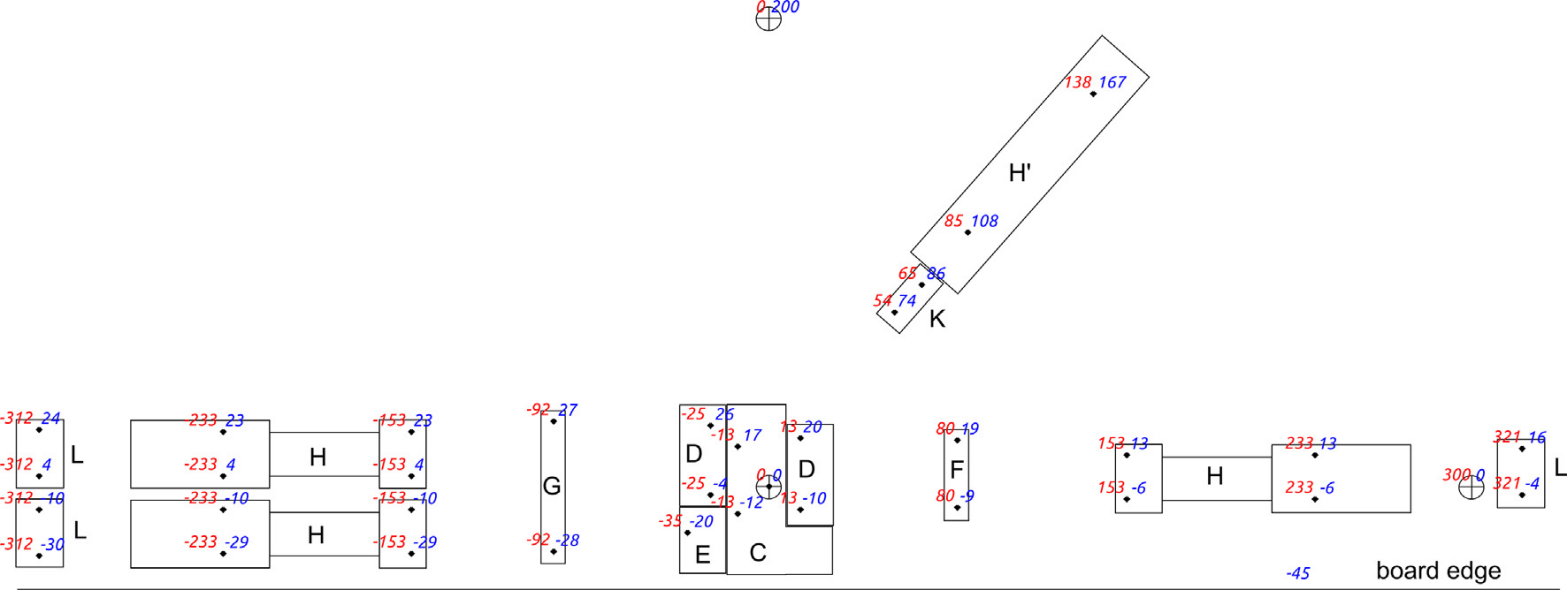
Assembly template for elements on the board. The letters and outlines of the elements correspond to the elements in Fig. 1, with the exception of letter H′, which corresponds to the coil platform and raises the valve driving actuator to the appropriate height. Black points indicate holes for mounting screws, the cross sign in the circle corresponds to the reference points, red is the X coordinate, and blue is the Y coordinate.

The coils and all holders should be attached to a mounting plate. The pistons should be inserted into the coils before installing them. A wooden board was used in this study for this purpose. The relative positions of all bolt holes are shown in Fig. 10, which can be found in pictures_movie download package at a 1:1 scale. It can be printed and used as a drilling template. The diameter of the bolt holes should not exceed 2 mm.

## Operation instructions

### Safety issues

The device described in the present study has movable elements, such as pistons and a valve wheel, to which a high force is applied within a short pulse, thus causing them to accelerate rapidly. Hands must be kept away during the sequence execution as these elements may cause injury. In addition, when the plungers move rapidly, the pressure of the solution in the system may be so high that it leaks and ejects the contents of the syringes in an uncontrolled manner or shoots off the ejection needle. Taking this into account, appropriate precautions should be taken, such as maintaining a proper distance from the device and wearing safety glasses.

**Operating hardware**

### Volume of solutions

First, the volumes and concentrations of the reagents should be selected. Considering the architecture of the device, each mixing is in the ratio of 1:1. This means that for double sequential mixing, the solutions from syringes 1 and 2 will be finally diluted four times in total, whereas the solution from syringe 4 only two times. Based on the magnetic actuators used in this project, the maximum volume in syringe 5 is 500 mL. Thus, the maximum volume for double sequential mixing is 125 µL for syringes 1 and 2 and 250 µL for syringe 4. This may be modified by some displacement in the mounting position of the syringe actuator 5 or by design alterations so that syringes with larger diameters can also be used. Because of the slight dilution of the solutions with the buffer located before the mixing sequence in the mixer channels, using a volume of solutions above 100 µL per syringe is recommended.

### Connecting the syringes to the mixing block

**Fig. 11:**
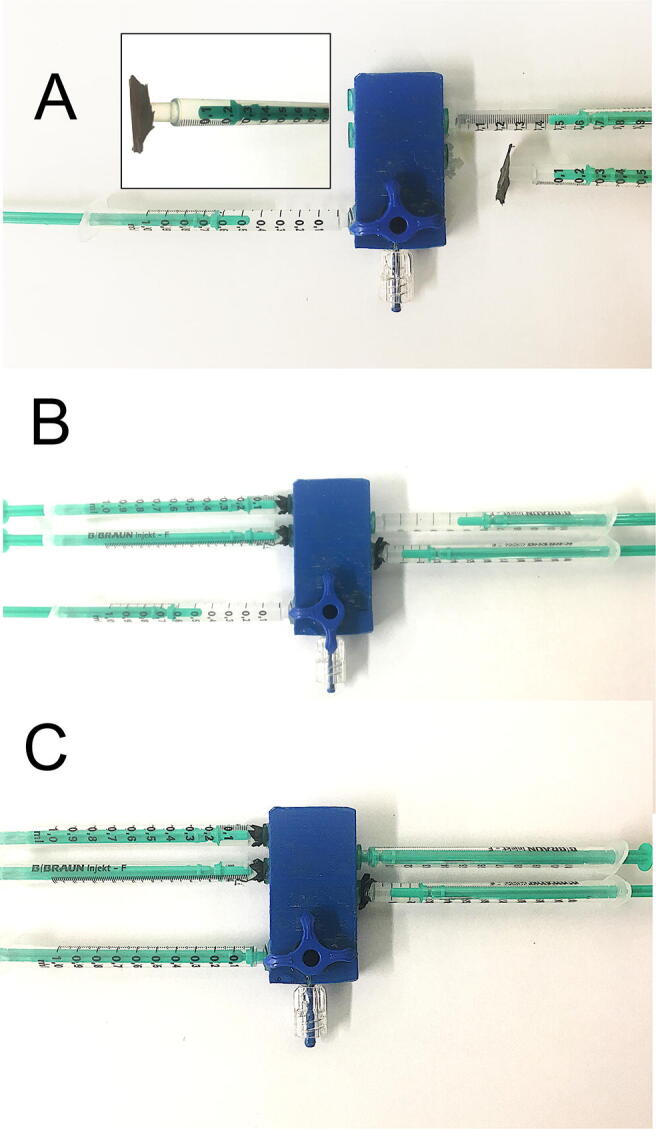
Preparation of the mixing block prior to the experiment shown in alphabetical order. A—set the valve in the stopped-flow mode (close outlet and syringe 3 connected with mixer), connect syringes 3 and 5 filled with buffer, and push the air out of the mixer with the buffer. Inset: prepare syringes with reagents: take the desired volume of reagents and put a thin foil at the end of the syringe outlet to prevent uncontrolled mixing of reagents after connection to the mixing block. B—set the valve to the full-open position and push out excess buffer from syringes 3 and 5. C—set the valve in the stopped-flow mode.

Before the syringes containing solutions are connected to the mixing block, the mixing channels should be washed with buffer. This can be carried out using a separate syringe. First, the valve should be set in the stopped-flow position and the syringes acting as the incubation chamber (syringes 5 and 3), which are filled with the buffer, should be attached. Then, any air in the mixer channels should be removed by gently pushing some of the buffer out of the mounted syringes. The remaining syringes have to be connected with the measured volume of reagents. A thin foil (e.g., stretch) can be used on the end of the syringe outlet to prevent uncontrolled mixing of the reagents. The foil breaks due to the pressure built up in the syringe when force is applied to the plunger. With all syringes connected, the valve has to be opened, the excess buffer removed from the incubation chambers, and the valve set in the proper position, which depends on the type of the experiment (usually stopped-flow mode position). The ejection needle needs to be attached to the mixer outlet. The procedure is shown in Fig. 11. The syringes used in this project are cheap disposable ones and must be replaced after each mixing sequence. This is because syringes deform under applied forces, resulting in leakages and increased friction of the plunger; moreover, the use of new syringes allows eliminating the risk of contamination of the solutions prior to mixing.

### Preparation for the shot

The assembled mixing block should be placed with syringes attached on the platform, and the syringes should be fixed to the holders. The diode has to be connected to the power supply, and the optic fiber has to be placed in the appropriate holder. The actuators’ piston should be moved toward the syringe so that it does not hit the syringe plunger at the beginning of the pulse. The piston blockers have to be adjusted to prevent excessive retraction of the plunger in syringes that are incubation chambers. The valve knob has to be placed on the three-way stopcock, and the string slack has to be selected by shifting the piston rod.

### Sequence programming

The device and the software controlling the magnetic actuators were previously described in Ref.[5]. Several factors must be taken into account while programming the sequences. First, the force applied to the piston should be optimized. For the pistons that simultaneously push two syringes, the force should be much greater than that used for pushing one syringe. In this case, it is recommend to apply pulses of full power. However, in the case of pistons that drive one syringe, the application of maximum force may impose too much pressure inside the syringe, which may lead to leakage, syringe breakage, or fluid cavitation; therefore, 60% of the power is a good starting point for the final optimization of power, if needed. Because the valve should be switched from the stopped-flow to the freeze-quench position at the shortest time possible, the power applied to the piston that turns the valve must be maximum. However, to prevent a strong blow to the lock, which in turn may cause a retraction, the initial maximum power should be decreased to approximately 25% a few milliseconds after the rotation of the valve is started. Using the power settings mentioned above, the total water flow rate is approximately 10 µL/ms per piston. It means that one piston needs 50 ms to push 500 µL of the total volume of the solution, i.e., 250 µL per syringe. It is worth noting that the flow values, depending on the force, may differ based on the viscosity of the solutions, small differences in the printing of the mixer, and the assembling of the actuators. Therefore, each experiment should be preceded by sequence optimization using buffers.

Another important issue regarding sequence programming must be taken into account. After the solution has been ejected from the syringes, it may flow into other syringes, which are prone to retraction. To avoid this, the retraction of syringe plungers can be temporarily blocked by applying a pulse to the syringe, and by slightly prolonging the duration of the blocking pulse than the duration of the pulse pushing the solutions. This means that the blocking pulse should start a few milliseconds before and stop a few milliseconds after the push pulse ends. An example sequence with these considerations taken into account is illustrated in Fig. 3.

## Validation and characterization

### Video recording

**Fig. 12:**
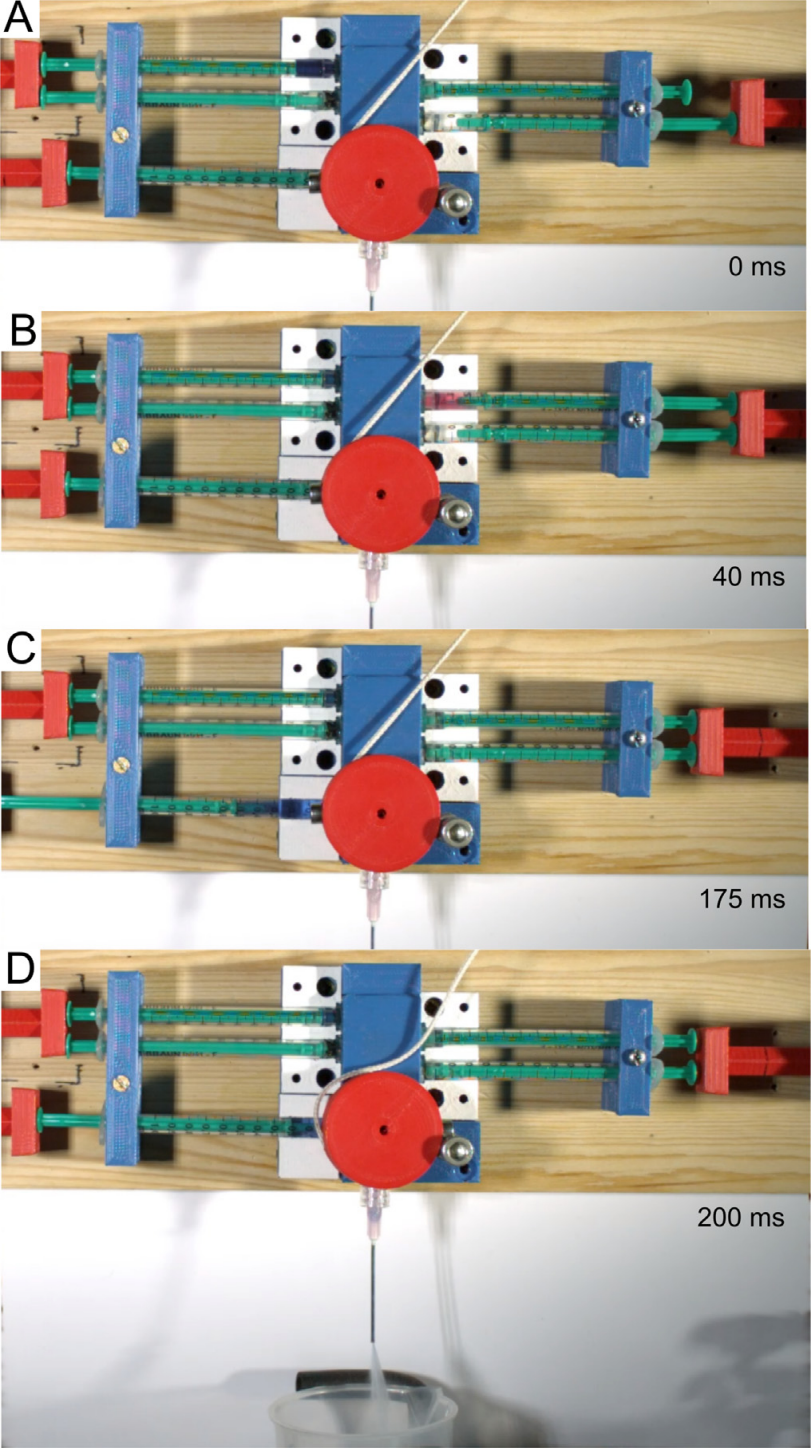
Selected frames from the fast-frame recording of double sequential mixing: A—immediately before starting the sequence, B—at the end of the first mixing, C—at the end of the second mixing, D—the mixture ejection after the valve switch. Note the color change of the dichlorophenolindophenol (DCPIP) solution in the syringes, which is due to the change in the pH of the solution resulting from mixing the DCPIP solution with an acid (B) and then with a base (C).

The device was validated in three ways. First, a sequence that enables sequential mixing of reagents and the combination of stopped-flow and freeze-quench experiments was recorded using a high-speed camera. The test was performed to monitor the process of mixing, valve rotation, and the piston movement. The recorded video shows the fastest sequence that was obtained using this system. The mixing of the solutions was monitored by a color change of dichlorophenolindophenol (DCPIP), which is blue at alkaline or red at acidic pH[6]. In the first step, a slightly alkaline DCPIP solution was mixed with a strong acid solution, which changed the color of the mixture from blue to red. After incubation, the red solution was subsequently mixed with a strong base, which restored the alkaline pH, making the mixture blue again. As the neutralization reaction is very fast (diffusion-limited process), any observation of the color change during the flow would suggest an incomplete mixing process. However, this is not the case, and the mixture that flows through the mixer into the syringes becomes immediately red already or blue after. This means that a simple meandering channel in the mixing block is sufficient to ensure the proper mixing of the solutions. The video also shows that for a correctly designed sequence, the pistons and the valve function as intended without retraction or deformation of the syringe plungers. The duration of the first mixing (2×125 µL) was approximately 35 ms, the second mixing (2×250 µL) was approximately 110 ms, the valve switching was approximately 10 ms, and the ejection from the fifth syringe was approximately 30 ms. The entire experiment was completed within 210 ms. Selected frames from the recording of the above-described mixing process are shown in Fig. 12.

**Fig. 13:**
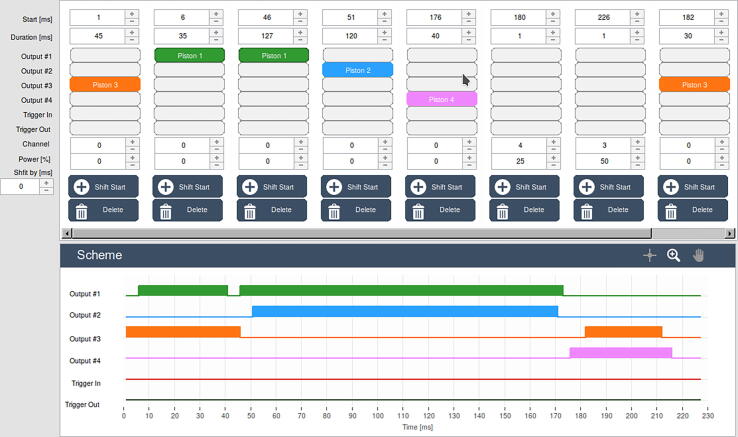
Screenshot of the sequence definition for the driver which was used to record the experiment shown in Fig. 12.

The recording containing the signature of the microseconds clock for each frame is included in the pictures_movie.zip download package. The times of syringe piston movements in the recording correspond very well to the respective times in the programmed sequence. The sequence used for the recordings is shown in Fig. 13. The initial power of the channels in all tests was 100%, except for channel 3 (syringe plunger 5), the initial power of which was 60%.

### Stopped-flow experiment – —simple chemical reaction

**Fig. 14:**
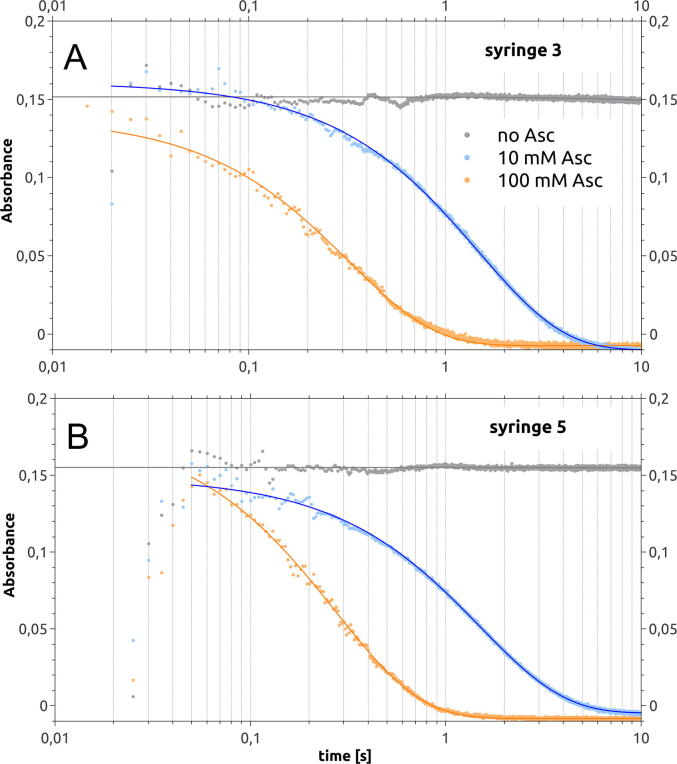
Kinetic curves of reduction of dichlorophenolindophenol (DCPIP) with ascorbate in the stopped-flow experiment monitored optically in syringe 3 (A) and syringe 5 (B). The absorbance is proportional to the oxidized form of DCPIP.

A simple chemical reaction was applied to test the device in the stopped-flow mode. The experiment was performed with each pair of syringes in the mixing block. In one experiment, reagents from syringes 1 and 2 (150 µL per syringe) were injected into syringe 3, in which the reaction was monitored. In the second independent experiment, reagents from syringes 3 and 4 were (250 µL per syringe) injected into syringe 5. The valve was in a fully closed position during these experiments. The syringes not used in the experiment were blocked by adjustable plugs to prevent their retraction from the zero-volume position. Syringe 4 was an exception, which could not be blocked at the zero-volume position when syringe 3 was used, so, it was filled with water and blocked at the position corresponding to the volume of 300 µL. The redox reaction of DCPIP with ascorbate in an alkaline pH was selected for the test since the reduction of DCPIP causes complete discoloration of blue DCPIP solutions[6]. The experiment was conducted with ascorbate at two concentrations, 10 and 100 mM, and without ascorbate. DCPIP concentration was 50 µM. The reaction progress was monitored using a TIDAS® S 500 MCS UV/VIS 1961 diode-array spectrometer in the wavelength range of 465–750 nm, and the integration time in the measurement was 5 ms. The spectrometer was connected to the mixing device using optic fiber. A white LED was used as the light source, which was placed in a dedicated slot in the syringe stabilizer. Synchronization of the optical measurement and the mixing sequence was ensured by the Trigger Out signal on the magnetic actuator controller. The sequence of pulses is presented in Table 3, Table 4. The results of the experiments are shown in Fig. 14.

**Table 3:** Pulse sequence used to observe reactions in syringe 3.

Start [ms]	1	3
Duration [ms]	1	25
Output 1		X
Output 2		
Output 3		
Output 4		
Trigger in		
Trigger out	X	
Channel	0	0
Power [%]	0	0

**Table 4:** Pulse sequence used to observe reactions in syringe 4.

Start [ms]	1	3
Duration [ms]	1	50
Output 1		
Output 2		X
Output 3		
Output 4		
Trigger in		
Trigger out	X	
Channel	0	0
Power [%]	0	0

In all time courses, the initial absorbance of the reaction mixture was virtually the same, equal to that of the DCPIP solution mixed with the buffer (absorbance = 0.15). This finding shows that the mixing of the reactants in the device is repeatable, provided that the syringes are precisely filled with respective solutions. Furthermore, the reaction time courses determined from the measurements at syringes 3 and 5 were similar. This confirms that the results of the experiment are not biased by the choice of the mixing system. Decay constants of absorbance are summarized in Table5.

**Table 5:** Summary of decay constants for measurements at syringes 3 and 4 for different ascorbate concentrations.

	Asc 10 mM	Asc 100 mM
Observation in syringe 3	1.49 s	1.47 s
Observation in syringe 5	0.274 s	0.32 s

It can be observed that the beginning of the interpretable data clearly corresponds to the end of the pulse. The outliers in the time course shortly after starting the measurement are due to the disturbances caused by the flow of solution into the measuring syringe. Because of these disturbances, the real measurement dead time is equal to the inflow time of the mixture.

### Combination of stopped-flow and freeze -quench experiments

To test the use of the device for the combination of stopped-flow and freeze-quench methods, the enzymatic reaction of cytochrome *c* reduction by cytochrome *bc*_1_ [7] was used. Syringes 1 and 2 contained substrates: 130 µL of decylubiquinol (1 mM) and oxidized cytochrome *c* (200 µM), respectively. A solution of 250 µL of the enzyme cytochrome *bc*_1_ at a concentration of 2 µM was placed in syringe 4. In the first step, the substrates were mixed as they must be kept separately to prevent a slow nonenzymatic reaction. Then, they were mixed with the enzyme, leading to the start of the reaction which was monitored. Cytochrome *c* reduction in syringe 5 was observed in two ways. First, the reaction was monitored by optical spectroscopy, in a similar way as DCPIP reduction, at the wavelength range of 500–600 nm to cover the cytochrome *c* spectrum. Second, the spin-lattice relaxation rate of the paramagnetic label attached to cytochrome *c*, which is sensitive to the cytochrome *c* redox state, was monitored. These measurements were performed by low-temperature pulse EPR spectroscopy, as described in Ref. [8] for frozen samples. The samples were prepared with the freeze-quench mode of the device by ejecting the reaction mixture from syringe 5 into the cold isopentane bath. The ejection also terminates the optical measurement. Since the freeze-quench method requires a certain number of sampling points, the experiment was performed 10 times with different incubation periods in syringe 5. The sequences of the pulses used in the experiment are shown in Table 6.

**Table 6:** Pulse sequence used in the test experiment combining stopped-flow and freeze-quench methods.

Start [ms]	0	10	500	505	510	575 + τ	579 + τ	580 + τ	592 + τ
Duration [ms]	40	30	69	2	60	40	1	50	1
Output 1		X	X						
Output 2					X				
Output 3	X							X	
Output 4						X			
Trigger in									
Trigger out				X					
Channel	0	0	0	0	0	0	4	0	4
Power [%]	0	0	0	0	0	0	25	0	10

**Fig. 15:**
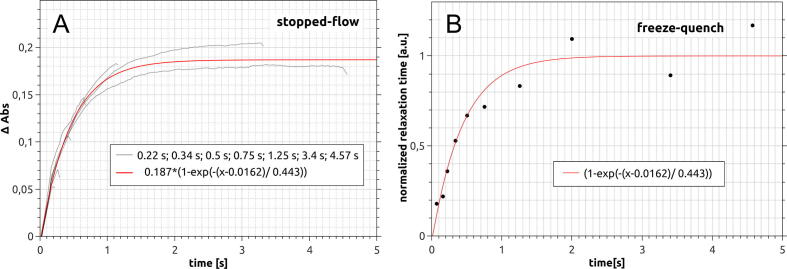
Enzymatic reduction of cytochrome *c* by cytochrome *bc*_1_ in the experiment with combined stopped-flow and freeze-quench modes. The amplitude of the curves is proportional to the concentration of the reduced form of cytochrome *c*. The reaction progress was monitored optically in syringe 5 (A), and the spin-lattice relaxation was measured by electron paramagnetic spectroscopy of frozen samples prepared by ejection of the reaction mixture to cold isopentane bath (B). The gray lines in panel A show the recorded time traces that end at different time points shown as closed circles in panel B. The red curve represents a function fitted to the experimental data.

The data obtained from optical measurements and using EPR spectroscopy are shown in Fig. 15.

The results obtained using the stopped-flow and freeze-quench methods correspond really well to each other. Although three curves could not be obtained, due to the appearance of a bubble through the optical path as a consequence of the presence of detergent, the time courses determined from optical measurements for different incubation times in the double-sequential-mixing experiment were reproducible.

The validation process showed that the developed device is indeed useful for chemical and enzymatic studies. The device was successfully tested in a stopped-flow experiment investigating simple chemical reactions as well as in a more advanced experiment with double sequential mixing of reagents combining stopped-flow and freeze-quench methods.

### CRediT authorship contribution statement

**Łukasz Bujnowicz:** Conceptualization, Writing – original draft, Investigation. **Rafał Pietras:** Validation, Investigation, Writing – review & editing. **Marcin Sarewicz:** Investigation, Writing – review & editing. **Artur Osyczka:** Supervision, Funding acquisition, Writing – review & editing.

## Declaration of Competing Interest

The authors declare that they have no known competing financial interests or personal relationships that could have appeared to influence the work reported in this paper.
